# Metagenomic next-generation sequencing to investigate infectious keratitis by *Corynespora cassiicola*: a case report

**DOI:** 10.3389/fmed.2023.1285753

**Published:** 2023-11-17

**Authors:** Shuo Xu, Shui Lu, Yan Gu, Hongjuan Sun, Minghui Ma, Yue Leng, Wenhui Liu

**Affiliations:** ^1^Department of Ophthalmology, Jiangnan University Medical Center, Wuxi, China; ^2^Department of Drug Clinical Trial Institution, Jiangnan University Medical Center, Wuxi, China; ^3^Department of Human Resources Division, The Affiliated Wuxi People’s Nanjing Medical University, Wuxi, China

**Keywords:** *Corynespora cassiicola*, fungal keratitis, keratoplasty, voriconazole, metagenomic next-generation sequencing

## Abstract

In this report, the case of a 65-year-old immunosuppressed female who presented with recurring redness and irritation in her right eye for 2 months is described. Ocular examination revealed conjunctival congestion, feather-like greyish-white corneal deep stromal infiltrate, white, floccular material sprawling from the anterior chamber angle and hypopyon. The *in vivo* confocal microscopy (IVCM) instantly confirmed fungal keratitis, and empirical antifungal therapy was thus administered. The patient exhibited therapeutic penetrating keratoplasty, however, due to the progression of infection and the lack of identified pathogens. The fungal isolate was identified as *Corynespora cassiicola* by metagenomic next-generation sequencing (mNGS) of the host cornea. The patient responded well to intensive conservative therapy and subsequent surgical therapy. To our knowledge, this case represents the first case of *C. cassiicola* infection from China, highlighting the emergence of a rare fungus that causes keratitis. Furthermore, mNGS has the capability to facilitate prompt identification and timely management of challenging ocular infections that are difficult to diagnose.

## 1 Introduction

As a result of filamentous fungi and yeast infection, fungal keratitis has been reported to account for 20–60% of all culture-positive corneal infections in tropical and subtropical locations (1). Approximately one and a half million cases of fungal keratitis occur worldwide each year, of which 100,000 progress to eye loss and 600,000 to blindness (2). The most relevant risk factors associated with fungal keratitis are vegetal-related trauma, topical corticosteroid use, contact lens use and systemic immunosuppression (3). Due to the limited antifungal treatment options, the inefficiency of antifungal therapy and the progressive nature of fungal keratitis (4), fungal keratitis is associated with poorer visual outcomes, a greater need for hospitalization and a higher incidence of complications than bacterial keratitis (2, 5). To provide efficient treatment and avoid complications, rapid and early diagnosis is crucial. However, conventional sampling and culture techniques are time-consuming, generally requiring 3–7 days, and have variably low culture positivity rates. Due to these substantial drawbacks, molecular techniques have been developed for infectious keratitis, including polymerase chain reaction (PCR), mass spectrometry and high-throughput sequencing approaches [targeted amplicon sequencing (16S rRNA in bacteria or 18S rRNA in fungi) and metagenomic sequencing (untargeted amplification of all genomic DNA)] (6, 7). In mNGS, all genomes in a given sample are sequenced simultaneously, resulting in a high resolution and effective detection of more species, such as viruses, eukaryotes and superkingdom archaea (8).

Herein, we report a case of fungal keratitis caused by *Corynespora cassiicola* (*C. cassiicola*), an aetiological agent of the Pleosporales order that is known to cause crop damage. As far as we know, there have been only two reported cases of ocular diseases caused by *C. cassiicola* (9, 10). This is the first study to use mNGS to identify *C. cassiicola*. The patient responded well to treatment consisting of intensive conservative therapy and subsequent surgical therapy.

## 2 Case report

We treated a 65-year-old Chinese female with rheumatoid arthritis and bladder cancer for recurrent redness and eye pain in the right eye for 2 months, which had been aggravated and accompanied by visual blurring in the past week. Prior to coming to our hospital, she had been diagnosed with uveitis at a local hospital and treated with topical and systemic corticosteroids, but her symptoms worsened. There was no prior history of contact lens use, diabetes mellitus or ocular disease, but there was a previous incidence of corneal trauma. The patient was diagnosed with penetrating corneal trauma for foreign body entry into the same eye 7 months earlier, and the patient’s symptoms had improved rapidly after treatment with systemic (levofloxacin, etimicin) and topical (0.3% gatifloxacin) antibiotics combined with intravitreal injections (vancomycin 1 mg/0.1 mL and ceftazidime 2.25 mg/0.1 mL). Intravitreal injections were prophylactically administered for infective endophthalmitis. Five years had passed since the patient underwent chemotherapy for bladder cancer, without recurrence to date.

**Figure 1:**
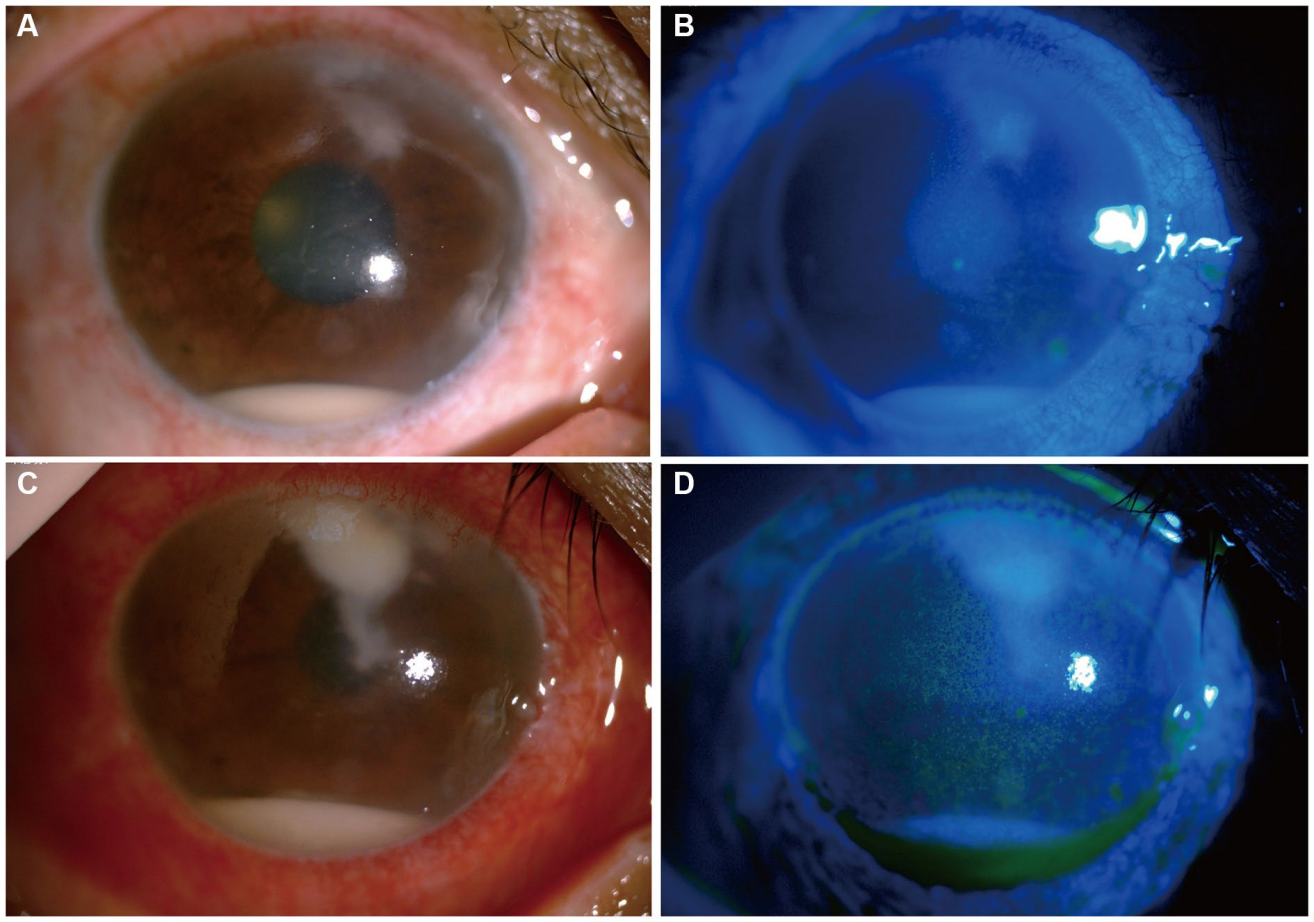
Changes in the patient’s corneal condition before penetrating keratoplasty. **(A,B)** Show slit-lamp and sodium fluorescein staining at the first visit, respectively. **(C,D)** Show slit-lamp and sodium fluorescein staining after 6 days of antifungal treatment, respectively.

**Figure 2:**
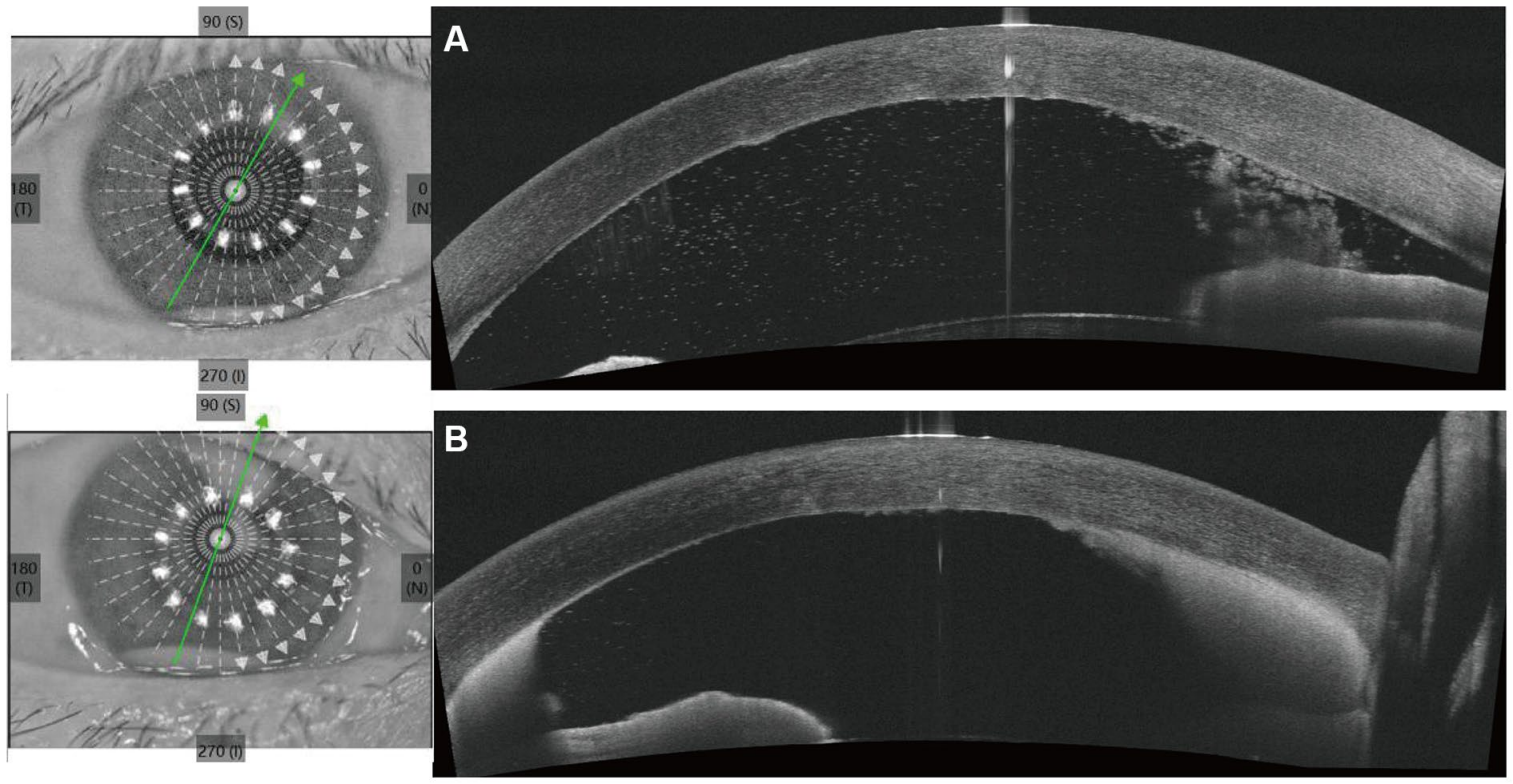
Anterior segment optical coherence tomography (AS-OCT) shows the changes of cornea and the anterior chamber angle before penetrating keratoplasty. **(A)** Shows the AS-OCT images at the first visit. **(B)** Shows the AS-OCT images after 6 days of antifungal treatment.

**Figure 3:**
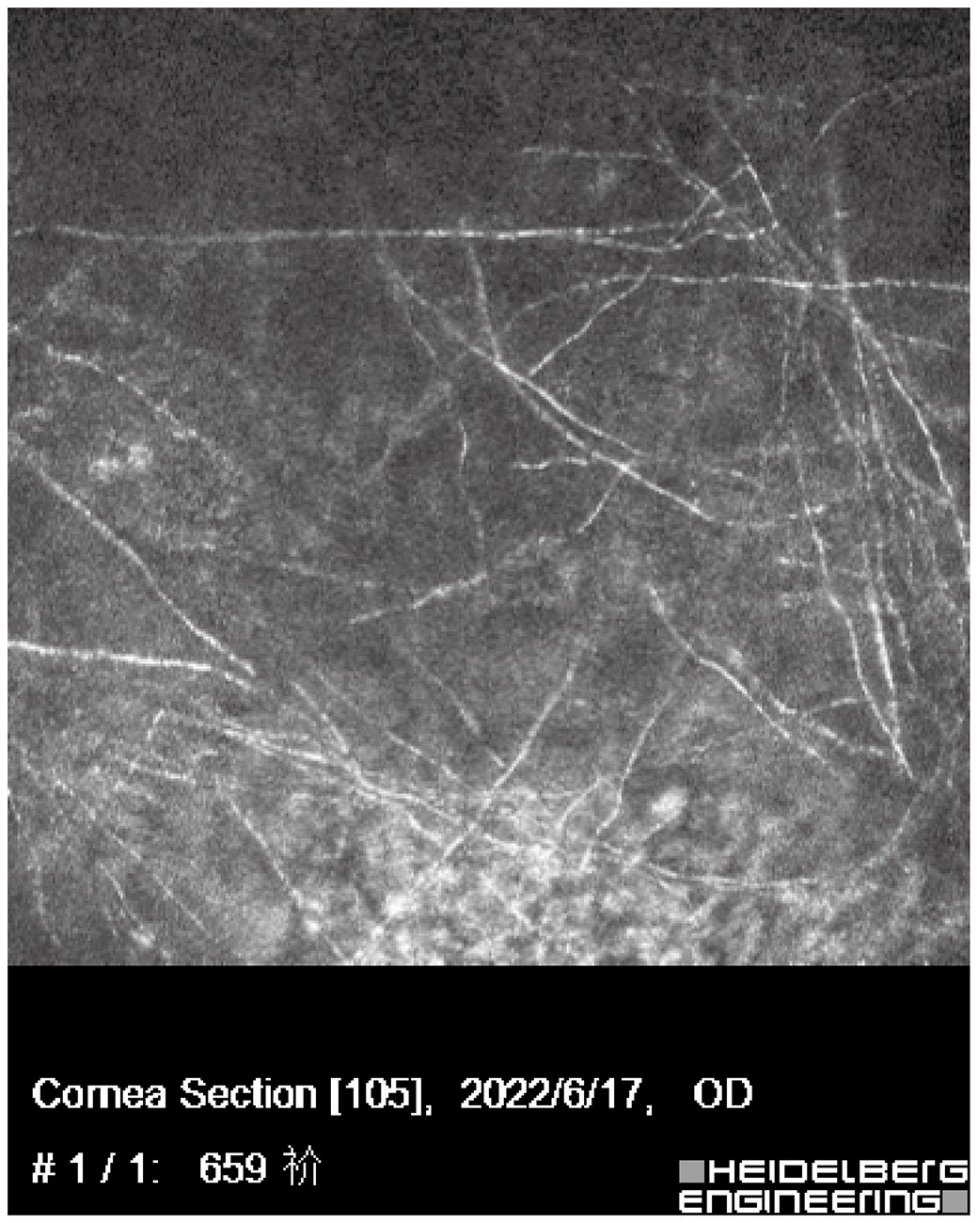
IVCM showed massive hyperlinear structures in the deep stromal layer which implies fungal infection.

Upon initial examination, the patient’s visual acuity was 6/20 in the right eye (OD) and 12/20 in the left eye (OS). The intraocular pressure was 15 mmHg OD and 14 mmHg OS. Slit-lamp anterior segment examination revealed conjunctival congestion, feather-like greyish-white corneal deep stromal infiltrate, white, floccular material sprawling from the anterior chamber angle and a 1.5 mm hypopyon (Figures 1A,B). The anterior chambers of both eyes were shallow. Both the maculas and optic nerves were normal during the fundus examination. Anterior segment optical coherence tomography (AS-OCT) showed infiltrated and opaque corneal stroma and an abundance of exudates attached to the endothelium (Figure 2A). The *in vivo* confocal microscopy (IVCM) images showed groups of hyperlinear structures in the deep stroma that were typical signs of fungal hyphae, as shown in Figure 3. No infections were found elsewhere in the body during a general medical examination. In addition, anti-HIV, anti-HCV, TPHA, HBsAg, ESR, IgG, and IgA levels, as well as rheumatoid factor and antinuclear antibody levels, were all negative, but the IgM was lower than normal. The patient was diagnosed with fungal keratitis and received systemic voriconazole at a dosage of 200 mg/day, topical 2% voriconazole eye drops once every 30 min and 0.5% levofloxacin eye drops 4 times a day. Despite the intense topical therapy, the infiltrate spread extensively to all corneal layers, forming a white floccular structure attached to the pupillary region, as shown in Figures 1C,D, 2B. Considering the progression and the continued lack of evidence of specific pathogens and to avoid affecting the vitreous body, therapeutic penetrating keratoplasty (TPK) with an 8.25 mm graft diameter was performed with complete macroscopic removal of the corneal lesion. Glycerol-preserved corneal tissue was used in the operation. After continuously washing the anterior chamber with 20 mL fluconazole (0.2 mg/1 mL), we removed the exudate membrane on the iris.

One sample from the excised host cornea was sent to Jiangnan University Medical Center Clinical Microbiology Laboratory for a fungal and bacterial culture, and another was sent to Beijing Giantmed Medical Diagnostics Lab for pathogen detection using mNGS. Total genomic DNA was isolated from the cornea by utilizing a QIAamp DNA Micro Kit (QIAGEN, Hilden, Germany). The DNA libraries were then constructed using the QIAseq™ Ultralow Input Library Kit (QIAGEN, Hilden, Germany), including fragmentation, end repair, adapter ligation, size selection and cleanup, and PCR enrichment and cleanup. Qualified libraries with different barcode labels were pooled and then sequenced on the NextSeq 550 platform (75 bp single-end reads) (Illumina, San Diego, United States). After the sequencing data were obtained and the adapter, as well as data exhibiting low quality, low complexity, and shorter reads (<35 bp) were filtered out, high-quality data were generated. Human reads were removed by mapping reads to the human reference genome using SNAP software. The remaining reads were finally aligned to the Microbial Genome Databases,^1^ which contain the whole-genome sequences of 8,472 viruses, 10,537 bacteria, 903 fungi and 288 parasites. The mNGS results identified *C. cassiicola*, with 421 sequences with a relative abundance of 99.76%. Cultures from corneal samples were negative, indicating neither bacteria nor fungi could be cultivated.

**Figure 4:**
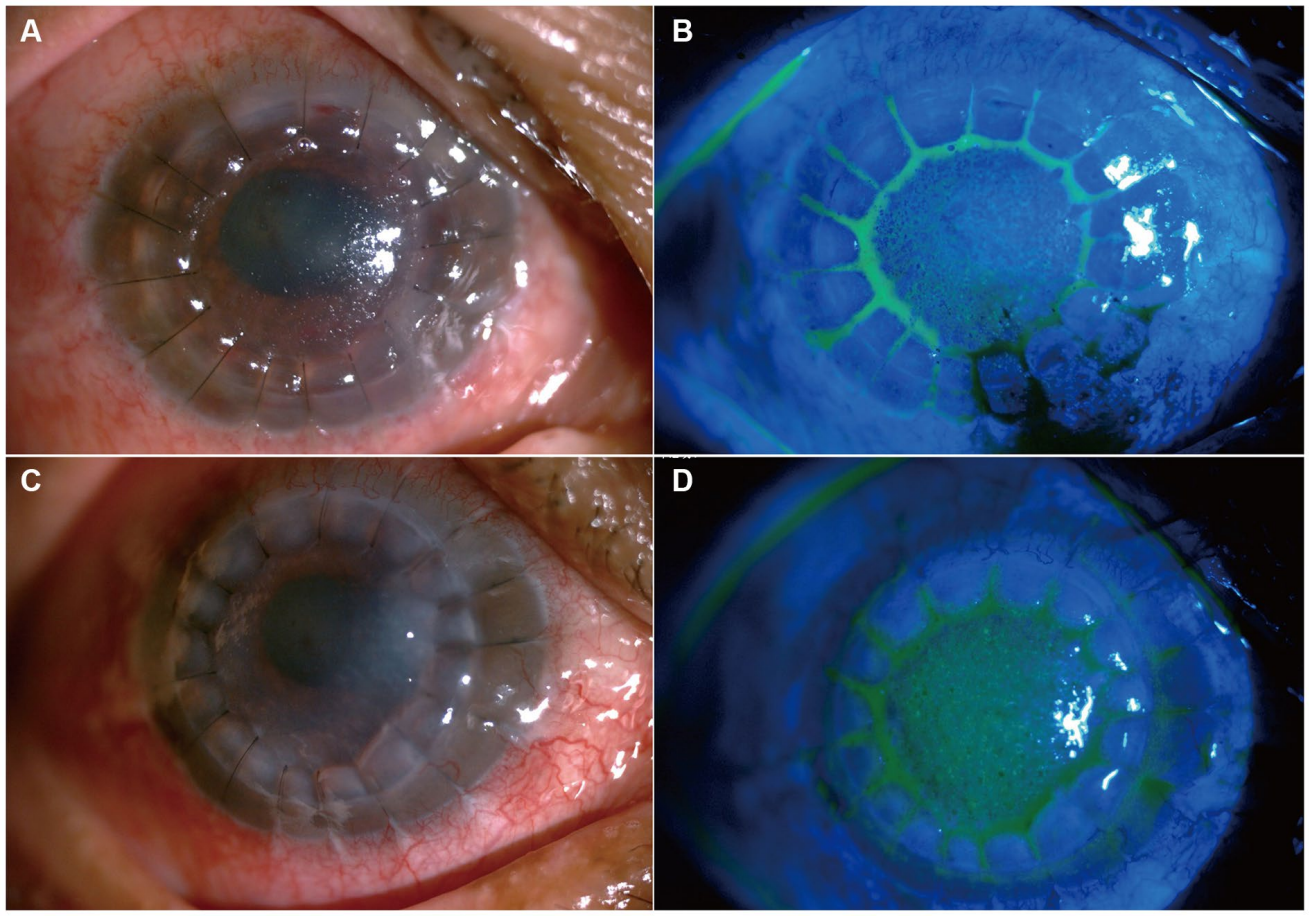
Slit lamp photo after penetrating keratoplasty. **(A,B)** Show slit-lamp and sodium fluorescein staining at the 6 days after keratoplasty, respectively. **(C,D)** Shows slit-lamp and sodium fluorescein staining two and a half months after keratoplasty.

**Figure 5:**
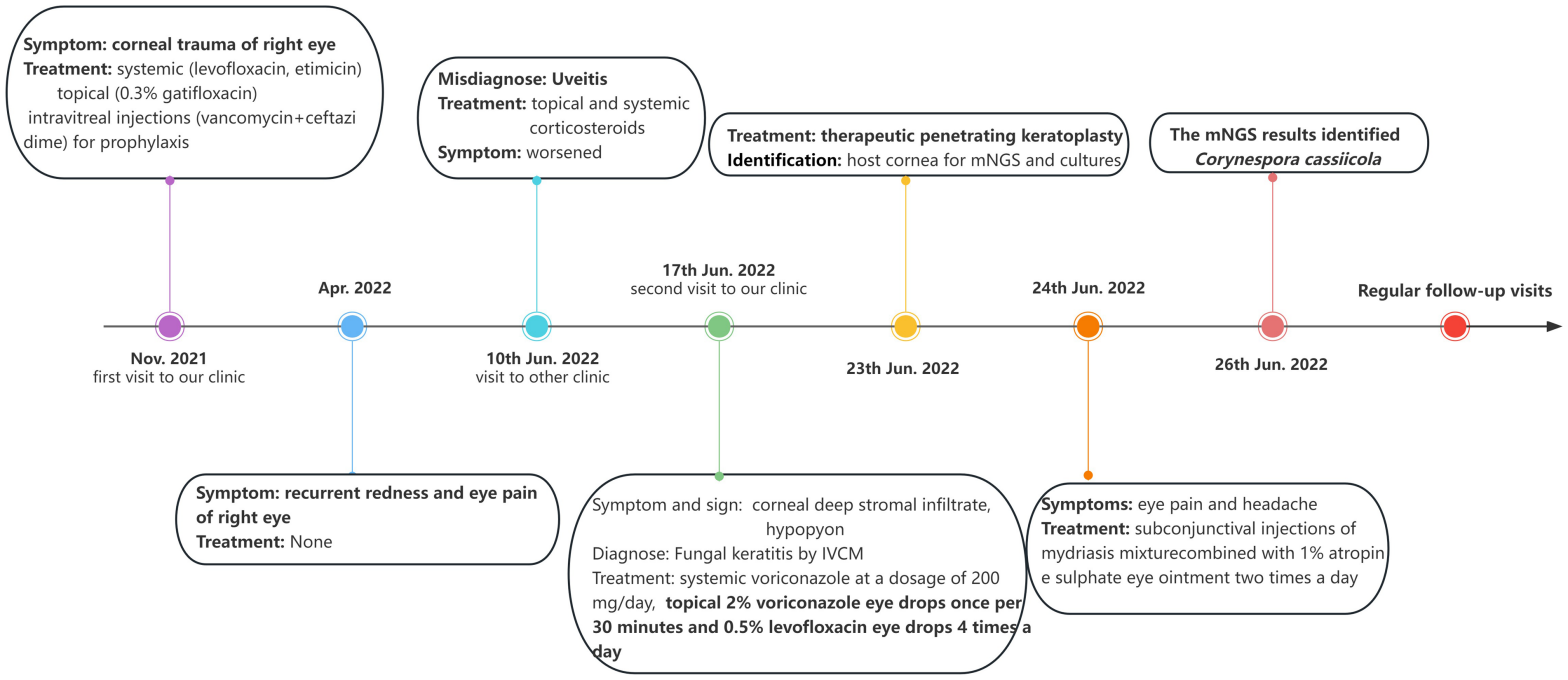
Timeline of the patient’ s disease progression and the corresponding treatment.

Considering the results of mNGS and previous treatment of *C. cassiicola* keratitis and endophthalmitis (9, 10), in the postoperative period, the patient was administered intravenous voriconazole (200 mg/dose per day), topical voriconazole 2% every 2 h, and ofloxacin (Tarivid) oculentum 0.3% and tropicamide 0.5% four times a day. The day after surgery, the patient developed symptoms of eye pain and headache. Considering that a large amount of inflammatory exudation was observed in the shallow anterior chamber during surgery and that anatomical alterations may change after therapeutic keratoplasty, we treated the patient with subconjunctival injections of a 0.1–0.3 mL mydriasis mixture (a mixture of 1% atropine sulphate injection 0.3 mL and 0.1% adrenaline injection 0.3 mL) combined with 1% atropine sulphate eye ointment two times a day to avoid pupillary block. Six days later, the intravenous voriconazole was discontinued. The patient was discharged, as depicted in Figures 4A,B. One month after surgery, the 2% voriconazole drops were tapered to six times a day. The patient was asymptomatic and had visual acuity of hand motion in the right eye. Following two and a half months, topical antifungal therapy was discontinued after ensuring no reactivation of the *C. cassiicola* infection by IVCM, and add 0.05% cyclosporin A for immunosuppressive therapy (Figures 4C,D). Timeline of her disease progression is shown in Figure 5.

## 3 Discussion

*C. cassiicola* is a member of the order Pleosporales in the class Hyphomycetes. First discovered on rubber trees in 1936, the fungus gained prominence after being isolated from a patient with mycetoma in 1969 (11). Numerous crops, such as papaya, rubber, cucumber, and tomato, have been damaged due to this aetiological agent (9). There have been only two reported cases of ocular infections caused by *C. cassiicola.* According to Gupta et al. (9), ITS sequencing of 28S rDNA confirmed the first Indian case of *C. cassiicola* endophthalmitis in a diabetic patient. Yamada et al. (10) reported the first case of *C. cassiicola* causing keratomycosis in a human host from Japan, which was identified by morphological characteristics and sequencing of the ITS region of 28S rDNA. The predisposing factors were numerous in our case, and the coexistence of corneal microdamage and immunosuppression should be considered. The patient had a history of ocular trauma 7 months prior, and the patient’s symptoms from this trauma improved rapidly after treatment with topical antibiotics. Although the patient was completely cured after this treatment, a relationship between this disease and the previous ocular trauma history cannot be ruled out. Furthermore, systemic and ocular immunosuppression are the main risk factors for ocular fungal infections (12), and the patient’s history of bladder cancer is also a principal risk factor.

IVCM examination of the cornea is a noninvasive technique that allows real-time identification of microbial keratitis pathogens, specifically filamentous fungal elements and Acanthamoeba cysts (2). A hyphae-like structure was observed under IVCM in our case, which can contribute to the initiation of rapid empirical antifungal therapy as soon as possible. Meanwhile, IVCM can be used to assess the recurrence or nonrecurrence of fungi during treatment. Given the progression and the continued lack of evidence of specific pathogens, we adopted mNGS, which is an unbiased high-throughput sequencing method that can theoretically quickly detect all pathogens in a clinical sample. However, microbiological investigations such as corneal scraping for culture and sensitivity testing remain the gold diagnostic standard for infectious keratitis. With an elevated trend of antibiotic or antifungal resistance, culture results play a vital role in the management of nonresponsive infectious keratitis cases or those that involving highly resistant rare species (7).

In the case described herein, the patient was initially misdiagnosed with uveitis at a local hospital. The glucocorticoid therapy-induced reduction in the host immune system promotes fungal keratitis development and progression. A consensus on the treatment modality and duration of *C. cassiicola* infection is currently lacking due to the dearth of reported cases and studies. Amphotericin B, itraconazole, terbinafine, voriconazole, posaconazole, micafungin or povidone-iodine, either as monotherapies or in combination, have been reported in the literature to adequately control infection (9). After Gupta et al. (9) diagnosed endophthalmitis, they immediately performed pars plana vitrectomy and administered intravitreal antibiotics at the end of the surgery. In the postoperative period, intravenous and topical antibiotics were administered until *C. cassiicola* was confirmed. The patient was subsequently started on oral voriconazole 200 mg twice a day, and the other topical treatments remained unchanged. At the time of the patient’s last follow-up, one year after presentation, his BCVA was 6/60 in OD, and there was no recurrence of fungal infection. Additionally, Yamada et al. (10) confirmed keratomycosis by corneal scrapings, and treatment was started with topical 1% voriconazole and 0.1% micafungin every hour, 5% pimaricin ointment five times daily, and 400 mg per day intravenous voriconazole. One month later, the intravenous voriconazole was discontinued and replaced by 400 mg/day oral voriconazole. The voriconazole was discontinued 1 month after discharge, and the antifungal drops were discontinued 3 months after discharge. No recurrence of the *C. cassiicola* infection was observed for 6 months. In our case, we also performed therapeutic corneal transplantation on the basis of systemic and local voriconazole treatment, and there was no recurrence of *C. cassiicola*. Voriconazole has become the most important antifungal, as it can be administered intravenously in addition to being applied locally. The application of voriconazole in *C. cassiicola* ocular infection cases has a therapeutic effect. Despite the effectiveness of antifungal therapy, due to this patient having severe corneal infections, therapeutic keratoplasty needed to be performed as early as possible to prevent anterior chamber involvement; however, if the corneal lesions were not completely removed, the risk of recurrence was high (13). After therapeutic keratoplasty, we thought that the lesions had been removed and the infection had been cured. However, in eyes with such intensive inflammatory reactions, anatomical alterations may develop, which in turn restricts the outflow of aqueous humour, causing increased intraocular pressure and possibly leading to therapy-resistant glaucoma (14).

In conclusion, the current case exhibited the importance of molecular methods in the diagnosis of human keratitis. High-throughput DNA sequencing can quickly provide an accurate diagnosis and avoid misdiagnosis with environmental colonizer fungi. However, microbiological investigations remain the irreplaceable method in infectious keratitis. Therapeutic keratoplasty is an effective treatment for severe infection. Our study is the first case report of *C. cassiicola* keratitis in an immunosuppressed patient, and this case was successfully managed.

## Data availability statement

The original contributions presented in the study are included in the article/supplementary material, further inquiries can be directed to the corresponding author.

## Ethics statement

The studies involving humans were approved by Jiangnan University Medical Center. The studies were conducted in accordance with the local legislation and institutional requirements. The participants provided their written informed consent to participate in this study. Written informed consent was obtained from the individual(s) for the publication of any potentially identifiable images or data included in this article.

## Author contributions

SX: Writing – original draft, Writing – review & editing. SL: Data curation, Writing – review & editing. YG: Data curation, Writing – review & editing. HS: Data curation, Writing – review & editing. MM: Investigation, Writing – review & editing. YL: Data curation, Writing – review & editing. WL: Writing – review & editing.

## Footnotes

^1^ftp://ftp.ncbi.nlm.nih.gov/genomes/
